# CD200R signaling inhibits pro-angiogenic gene expression by macrophages and suppresses choroidal neovascularization

**DOI:** 10.1038/srep03072

**Published:** 2013-10-30

**Authors:** Shintaro Horie, Scott J. Robbie, Jian Liu, Wei-Kang Wu, Robin R. Ali, James W. Bainbridge, Lindsay B. Nicholson, Manabu Mochizuki, Andrew D. Dick, David A. Copland

**Affiliations:** 1 Academic Unit of Ophthalmology, School of Clinical Sciences, University of Bristol, Bristol, UK; 2 Department of Ophthalmology, Graduate School of Medical and Dental Sciences, Tokyo Medical and Dental University, Tokyo, Japan; 3 Department of Genetics, UCL Institute of Ophthalmology, London, UK; 4 School of Cellular and Molecular Medicine, University of Bristol, Bristol, UK; 5 These authors contributed equally to this work.

## Abstract

Macrophages are rapidly conditioned by cognate and soluble signals to acquire phenotypes that deliver specific functions during inflammation, wound healing and angiogenesis. Whether inhibitory CD200R signaling regulates pro-angiogenic macrophage phenotypes with the potential to suppress ocular neovascularization is unknown. CD200R-deficient bone marrow derived macrophages (BMMΦ) were used to demonstrate that macrophages lacking this inhibitory receptor exhibit enhanced levels of *Vegfa, Arg-1* and *Il-1β* when stimulated with PGE_2_ or RPE-conditioned (PGE_2_-enriched) media. Endothelial tube formation in HUVECs was increased when co-cultured with PGE_2_-conditioned CD200R^−/−^ BMMΦ, and laser-induced choroidal neovascularization was enhanced in CD200R-deficient mice. In corroboration, signaling through CD200R results in the down-regulation of BMMΦ angiogenic and pro-inflammatory phenotypes. Translational potential of this pathway was investigated in the laser-induced model of choroidal neovascularization. Local delivery of a CD200R agonist mAb to target myeloid infiltrate alters macrophage phenotype and inhibits pro-angiogenic gene expression, which suppresses pathological angiogenesis and CNV development.

Severe and sudden visual impairment in patients with age-related macular degeneration (AMD) characterized by choroidal neovascularization (CNV), occurs where pathological angiogenesis extends from choriocapillaris into the subretinal space and retina^1^,^2^. Recent revised clinical definitions of AMD describe early, intermediate and late disease phases, based on the appearance and size of drusen formation at the RPE/Bruch's membrane interface^3^. Drusen are immunologically active deposits containing oxidative lipids, complement and other potential immune activating components that develop in response to environmental stress and altered tissue homeostasis^4^,^5^,^6^. Drusen are considered to be precursors of advanced disease, namely RPE dysfunction, death and ultimately geographic atrophy. Whilst the precise mechanisms that initiate the dominant VEGF drive of CNV in AMD remain elusive, the switch towards the neovascular form of disease may occur during any of the clinical stages of AMD.

Although patients with AMD do not display signs of overt ocular inflammation, it is recognized that both innate and adaptive immune responses contribute to the pathogenic pathways in AMD^7^. The accumulation of myeloid cells, especially macrophages adjacent to and within drusen occurs in all clinical stages of disease, but in particular a larger number are often present at the time of CNV^1^,^4^. The function and phenotype of macrophages is conditioned by signals encountered within the tissue microenvironment, and in mouse, the paradigm of M1 and M2 macrophages has been studied with respect to angiogenesis^8^,^9^. Classical activation generates M1 macrophages (nitric oxide synthase 2, NOS2^+^) which have pro-inflammatory functions, whereas alternatively activated M2 macrophages (Arginase-1, Arg-1^+^) confer responses related to wound healing, and are capable of generating VEGF and promoting angiogenesis. However, pathological angiogenesis is observed most commonly in the presence of M2 macrophages^10^,^11^ that generate VEGF either through conditioning by Th2 cytokines, IL-4 or IL-13^8^, or via PGE_2_ signaling^12^.

Despite conflicting evidence from studies investigating potential mechanisms whereby macrophage phenotype contributes to CNV progression, the general consensus is that activated macrophages, on the background of a highly complex and dynamic ocular microenvironment are central to the process^6^,^13^,^14^,^15^,^16^,^17^. The role of macrophages driving a VEGF-dependent angiogenic response is supported by recent evidence from studies using the laser-induced CNV model that show early initiation of choroidal angiogenesis is dependent upon macrophage phagocytosis of damaged RPE components which elicits an Arg-1^+^ VEGF^+^ M2 phenotype^18^. These data support the view that targeted interventions that modulate myeloid activation or protect against RPE death may prove beneficial for prevention of these early events that trigger CNV.

The CD200:CD200R interaction provides a normal homeostatic control mechanism by which CD200 binds to the inhibitory CD200R receptor expressed predominantly on cells of myeloid lineage, to limit potentially damaging pro-inflammatory responses^19^,^20^. CD200 is expressed abundantly within the eye, on retinal vascular endothelium, neurons and RPE, and is known to regulate myeloid cell activation^21^,^22^. Furthermore, previous studies demonstrate that modulating macrophage responses through CD200R with a receptor specific agonist, DX109, can suppress activation and protect against tissue damage in inflammatory ocular environments^23^.

The purpose of the present study was to interrogate whether CD200R signaling regulates the proangiogenic macrophage phenotype (Arg-1^+^VEGF^+^) pivotal to initiation of angiogenesis, and whether targeting the inhibitory CD200R could modulate early infiltrating macrophage phenotype and function, thereby influencing the clinical outcome of disease.

## Results

### CD200R-deficient macrophages demonstrate a pronounced proangiogenic phenotype following PGE_2_ stimulation

Vascular endothelial growth factor (VEGF) is critical to the process of vascular remodeling during tissue repair and wound healing responses following inflammation, but under pathological conditions VEGF-mediated angiogenesis may cause tissue damage, as observed in AMD. Alternatively activated macrophages, characterized by canonical arginase-1 (Arg-1) expression, also generate VEGF in response to various environmental signals, including Th2 cytokines (IL-4, IL-13)^8^,^9^ as well as immune-modulatory compounds such as PGE_2_^12^. The inhibitory CD200R, expressed predominantly by cells of myeloid lineage provides a normal homeostatic control mechanism that serves to limit cell activation^19^,^23^.

**Figure 1:**
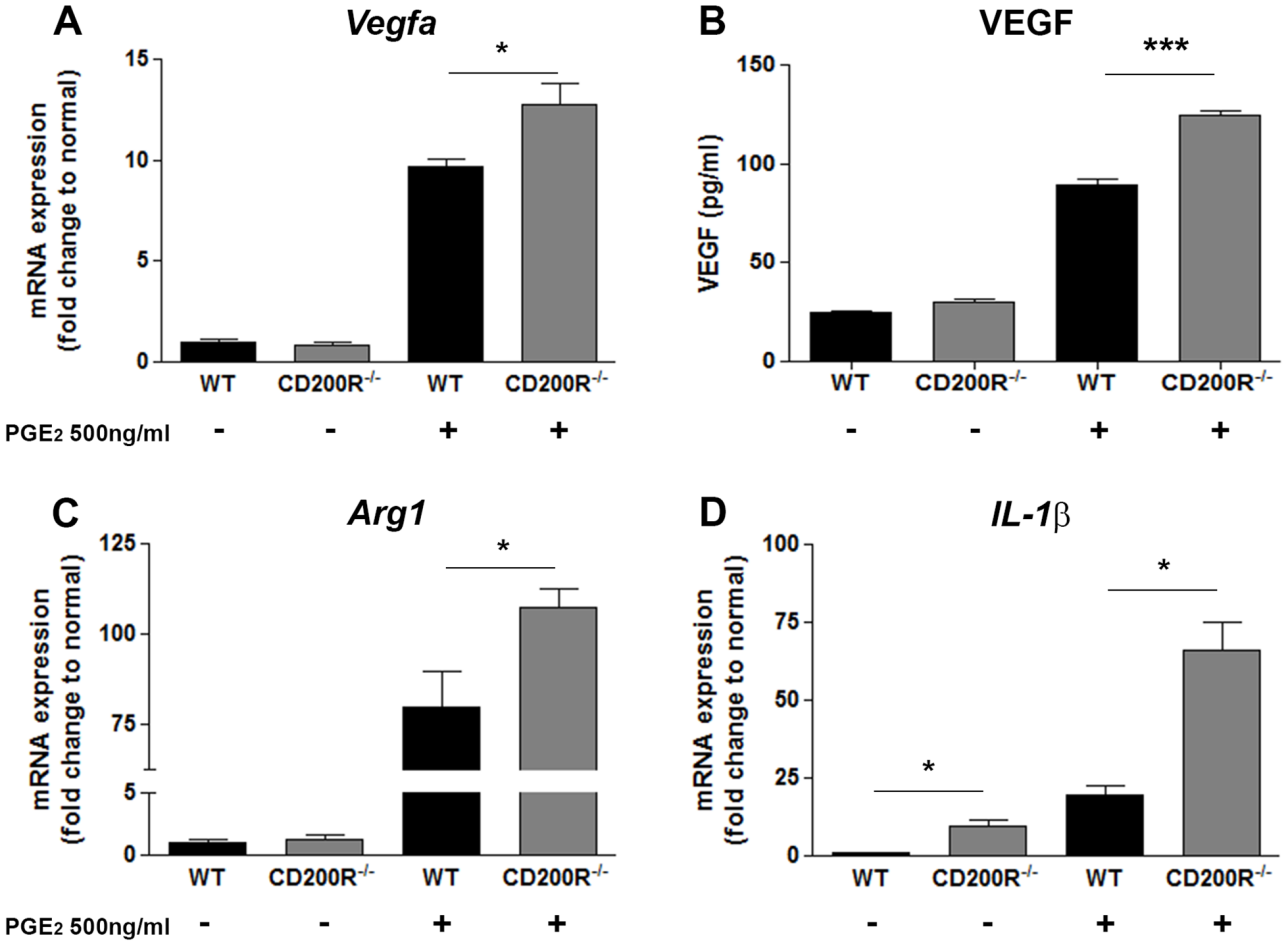
CD200R-deficient macrophages demonstrate a pronounced proangiogenic phenotype following PGE_2_ stimulation. (A), (C), (D) *Vegf, Arg1* and *Il1β* mRNA expression, respectively, determined by quantitative RT-PCR; (B) VEGF protein production from cell supernatants derived from WT and *CD200R^−/−^* macrophages stimulated by PGE_2_ for 6 h, as determined by ELISA. Messenger RNA level was normalized to 18srRNA. Data are presented as mean ± SEM, n = 3. *P < 0.05, and ***P < 0.0005 between two groups.

To determine whether and in what context CD200R signaling influences M2 macrophage activation and phenotype we generated bone marrow derived macrophages (BMMΦ) from C57BL/6J (wild-type; WT) and CD200R-deficient (CD200R^−/−^) animals and cultured them in the presence of PGE_2_ for 6 hours, before analyzing for gene expression. Previously, it has been shown that PGE_2_ stimulation of WT macrophages results in increased *Vegfa* and *Arg1* gene expression^12^. Our initial data not only confirmed this response but also demonstrated that *Il-1β* mRNA levels are significantly upregulated, following a similar dose-dependent response (Supplementary Figure 1). The greatest induction of gene expression was achieved when macrophages were stimulated with 500 ng/ml PGE_2_, and this concentration was therefore used for subsequent *in vitro* assays. Analysis of *CD200R^−/−^* macrophages stimulated with PGE_2_ revealed these cells to have a more pronounced proangiogenic response, with significantly elevated levels of *Vegfa, Arg1 and Il-1β* mRNA when compared to WT (Figure 1A, C, D). The basal expression of IL-1β from *CD200R^−/−^* macrophages was also significantly greater than that from WT cells. Furthermore, PGE_2_ stimulation resulted in increased VEGF production in *CD200R^−/−^* macrophages (Figure 1B). Levels of *IL-18* mRNA expression were also analyzed, and although elevated (2-fold change increase) in PGE_2_-stimulated CD200R^−/−^ macrophages, the overall response was markedly reduced when compared to *IL-1β* induction (60-fold change) (Supplementary Figure 2).

### Lack of CD200R signal results in enhanced macrophage VEGF expression in response to RPE-derived PGE_2_

**Figure 2:**
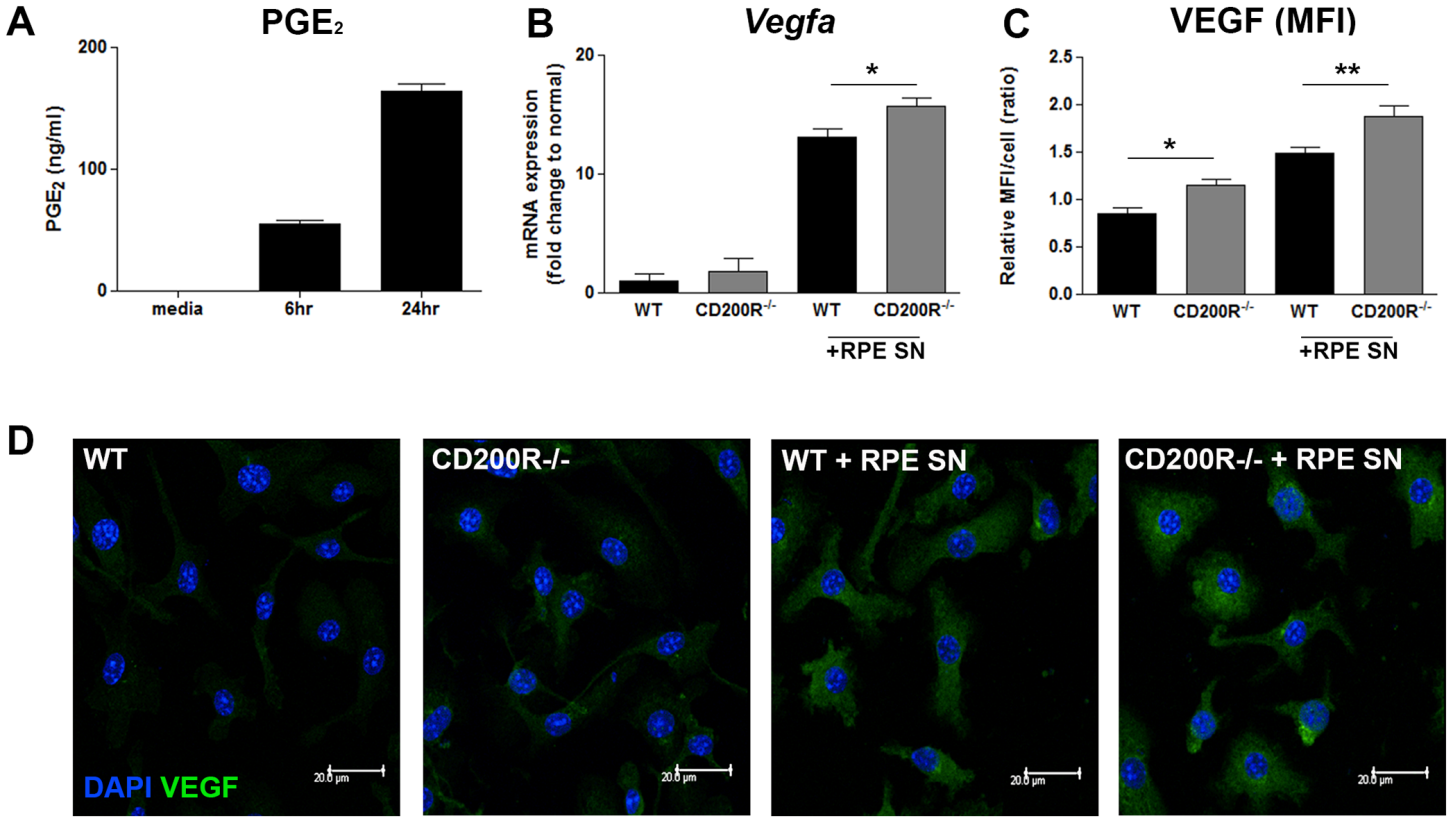
Lack of CD200R signal results in enhanced macrophage VEGF expression in response to RPE-derived PGE_2_. (A) PGE_2_ secretion from murine B6RPE-07 cells cultured for 6–24 hr was determined by ELISA. (B) *Vegf* mRNA expression from BMMΦ cultured for 6 hr in RPE conditioned media collected after 24 hr. 18srRNA was used as internal control. (C) Relative mean fluorescent intensity (MFI) of VEGF expression in WT or CD200R1^−/−^macrophages. (D) Representative confocal images of BMMΦ cultured in RPE supernatants or control media for 24 h. Cells were fixed and permeabilized before immuno-staining with anti-VEGF antibody. Scale bar: 20 μm. Data are presented as mean ± SEM, n≧3. *P < 0.05, **P < 0.005 between groups.

We have shown that CD200R deficiency drives bone marrow-derived M2 macrophage activation in the context of PGE_2_-mediated *Vegfa*, *Arg* and *IL-1β* expression. In relation to the chorioretinal complex of the eye, retinal pigment epithelium (RPE) cultures secrete PGE_2_, as demonstrated in studies that use both murine primary cultured RPE cells and the human ARPE-19 cell line^24^,^25^. On this basis, we replicated conditions in the subretinal microenvironment to determine how macrophages respond to RPE-derived PGE_2_, and whether CD200R signaling had any role in modulating this. Utilizing the recently characterized murine RPE cell line (B6-RPE07)^26^, we examined both PGE_2_ secretion from cultured RPE cells, and the effect of supernatant derived from these cells on macrophage phenotype. Conditioned media from cultured B6-RPE07 cells, contains high levels of PGE_2_ after 24 hrs (Figure 2A), and addition of this cell supernatant to WT BMMΦ resulted in upregulated *Vegfa* mRNA expression as compared to media controls (Figure 2B). Moreover, *Vegfa* mRNA and VEGF expression, as confirmed by analysis of confocal microscopy images, were elevated in *CD200R^−/−^* BMMΦ when compared to WT (Figure 2B–D).

### The enhanced pro-angiogenic phenotype of CD200R-deficient macrophages promotes angiogenesis

**Figure 3:**
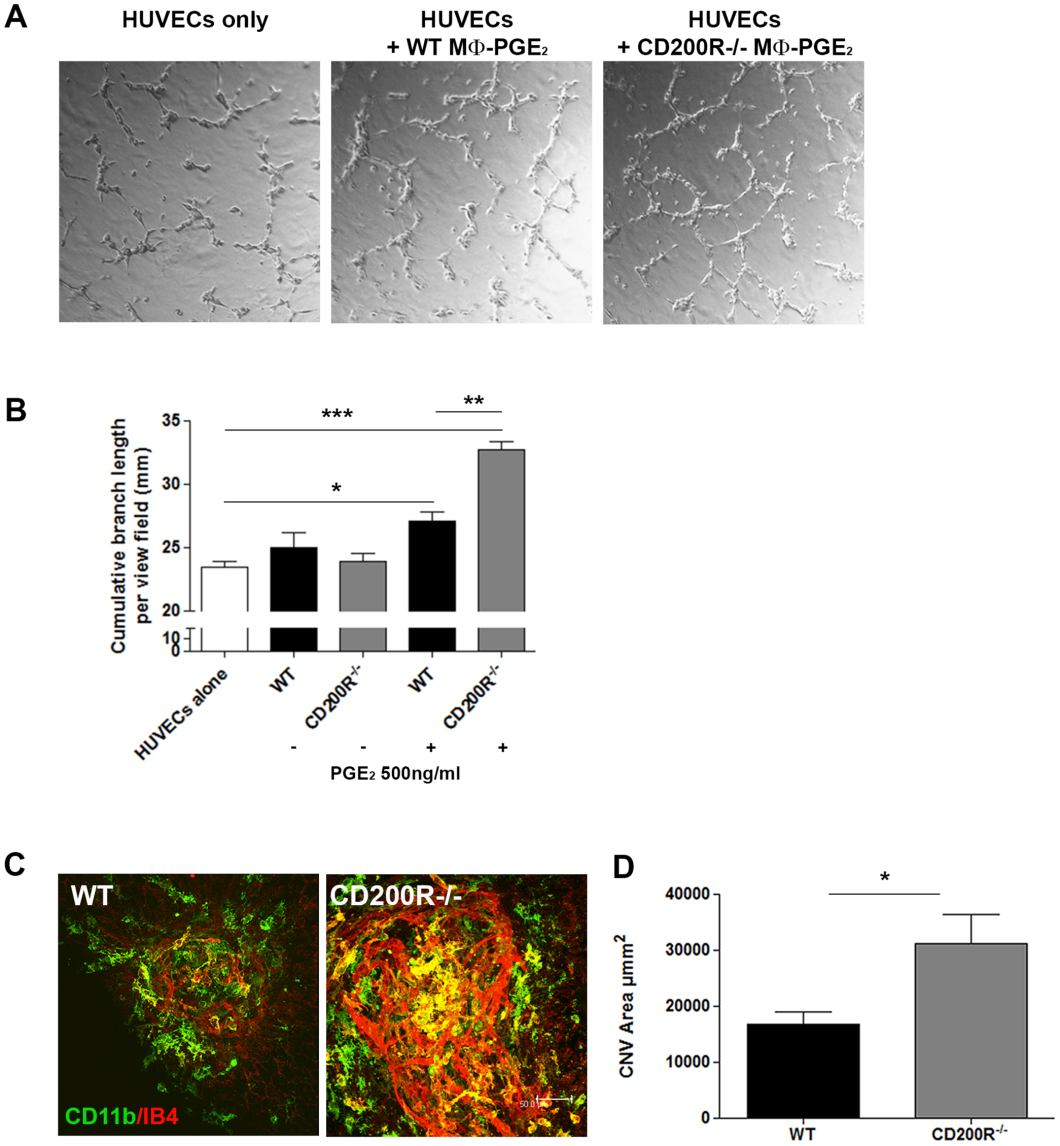
Enhanced pro-angiogenic phenotype of CD200R-deficient macrophages promotes angiogenesis. (A) Representative images of HUVEC cells cultured alone, or in the presence of WT or *CD200R*^−/−^ macrophages stimulated with PGE2 for 24 h in advance. (B) Cumulative branch length per field of HUVEC cells cultured alone, or in the presence of WT or *CD200R^−/−^* macrophages stimulated, or not, with PGE_2_. Data are presented as mean ± SEM, n = 3, *p < 0.05; **p < 0.005; ***p < 0.0005 between groups. (C) Representative confocal images of RPE/choroid flat-mounts from WT and *CD200R^−/−^* mice at day 7 post-laser application, were immunostained with CD11b (green) and Isolectin B4 (IB4) (red). (D) Mean CNV area calculated from confocal images. Data presented as mean ± SEM, n = 26 lesions for each strain; *p < 0.05.

Having established an elevated VEGF response in PGE_2_-stimulated CD200R-deficient macrophages, we sought to determine whether this phenotype translated to enhanced pro-angiogenic functions. Using the HUVEC angiogenesis assay^27^, we examined whether co-culture with PGE_2_ stimulated WT or *CD200R^−/−^* macrophages influenced endothelial cell tube formation (Figure 3A, B). The addition of WT macrophages stimulated with PGE_2_ led to increased HUVEC tube formation measured as the cumulative branch length per field of view, however tube formation following co-culture with PGE_2_-conditioned *CD200R^−/−^* BMMΦ was significantly greater demonstrating the requirement for intact CD200R signaling to suppress macrophage pro-angiogenic function.

The laser-induced model of choroidal neovascularization (CNV), employs laser photocoagulation to rupture Bruch's membrane (a pentalaminar configuration of elastin and collagen fibres located under the RPE) and stimulate a neovascular response that is associated with acute RPE dysfunction, cell loss and dysregulated immune responses^28^,^29^. Given the evidence for a central role of macrophages in driving CNV^17^,^30^, we examined whether lack of CD200R signaling and an associated elevated macrophage VEGF response would influence choroidal neovascularization and angiogenic development in this model. Laser-induced CNV was evaluated in WT C57BL/6J and *CD200R^−/−^* mice, and CNV lesions assessed at 7 days following laser application, the time when angiogenesis and neovascular membranes are established. Analysis of CNV lesion sizes, based on IB4 immunostaining of RPE/choroid flatmounts, revealed that lesions in *CD200R^−/−^* mice were significantly larger compared with WT animals (Figure 3C & D).

### Promoting CD200R signaling can suppress pro-angiogenic macrophage gene expression

The current results demonstrate that in the absence of CD200R signaling, both the phenotype and proangiogenic activity of macrophages are altered, suggesting that engagement of CD200R may offer the potential to influence macrophage activity and CNV development. Such an approach using an agonist monoclonal antibody DX109 has been successful in suppressing cell activation and disease severity in several experimental disease models^19^,^23^. We therefore examined whether engagement of CD200R, using the DX109 agonist, could modulate the PGE_2_-induced pro-angiogenic macrophage gene signature. In these experiments, WT BMMΦ were pre-incubated with DX109 or control mAb for 2 hr before PGE_2_ stimulation. Promoting CD200R signaling did not alter macrophage phenotype, with levels of Vegfa, Arg-1 and IL-1β expression remaining unchanged (Supplementary Figure 3).

**Figure 4:**
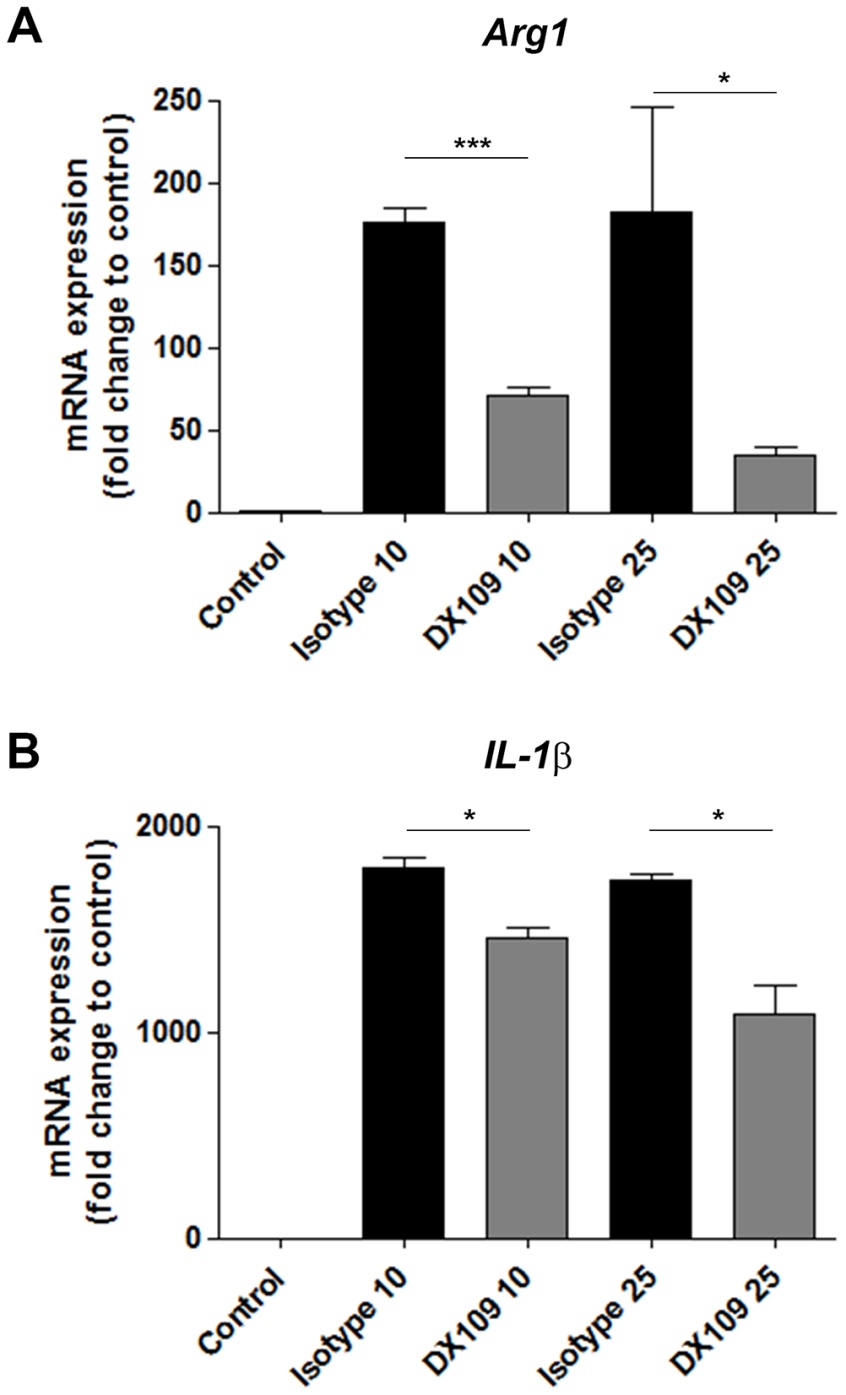
Promoting CD200R signaling suppresses pro-angiogenic gene expression profile of macrophages. Using an adapted *in vitro* phagocytosis assay, necrotic RPE cells prepared from B6-RPE07 cells by heating at 95°C for 15 minutes, were added to BMMΦ that were pre-incubated for 2 hours with 10- or 25 μg/ml DX109 or isotype control mAb. BMMΦ were washed and collected after 24 hr incubation with necrotic RPE, and RNA extracted for RT-qPCR analysis to determine *Arg-1* and *Il-1β* gene expression. *Gapdh* was used as a normalizing control. Data are presented as mean ± SEM, n = 3. *p < 0.05; ***p < 0.0005.

Central to CNV development in the laser model are CCR2^+^ macrophages^31^. We have recently shown that recruited macrophages are conditioned via uptake of damaged RPE components toward a proangiogenic Arg-1^+^ VEGF^+^ phenotype, and the selective depletion of these cells suppresses CNV development^18^. Therefore we examined whether DX109 could modulate the pro-angiogenic gene expression of macrophages conditioned through the uptake of damaged RPE components. Using an adapted *in vitro* phagocytosis assay, WT BMMΦ were pre-incubated with DX109 or an isotype control for 2 hrs before the addition of heat-induced necrotic mouse B6-RPE07 cells. After 24 hr, the effect of CD200R engagement on gene expression was assessed. DX109 treatment (10- and 25- μg/ml) was able to inhibit the expression of both *Arg-1* and *IL-1β* when compared with isotype mAb (Figure 4), but this effect did not extend to suppressing *Vegfa* expression (Supplementary Figure 4).

### Promoting CD200R signaling can suppress pro-angiogenic gene expression and CNV development

**Figure 5:**
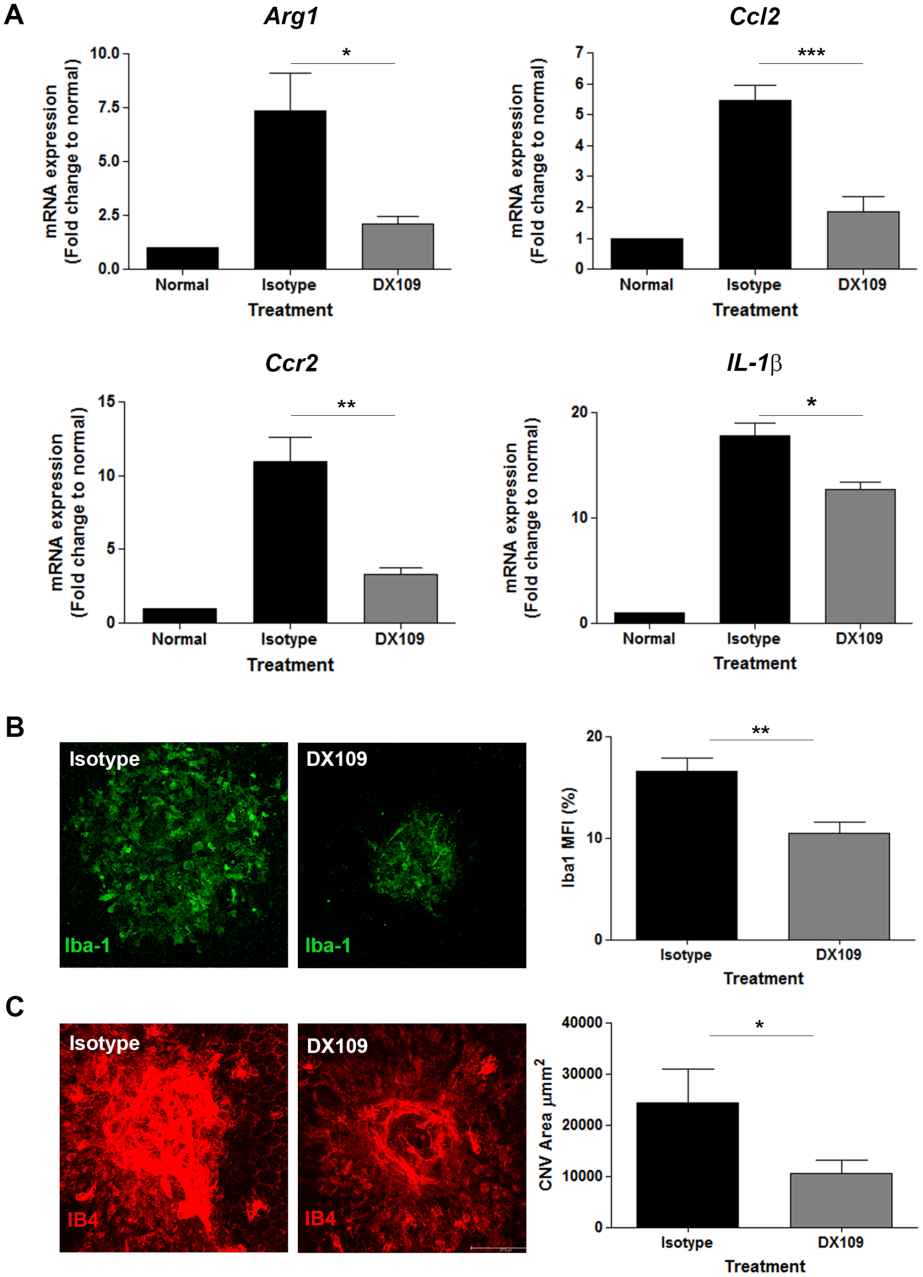
DX109 treatment alters the proangiogenic gene signature of CNV and reduces infiltration of Iba-1^+^ macrophages. Intravitreal administration of DX109 or isotype control mAb was performed immediately following laser induction of CNV in C57BL/6J wild-type mice. (A) RT-pPCR analysis for *Arg-1*, *Ccl2*, *Ccr2* and *Il-1β* gene expression in RPE/choroid tissue collected at day 3 post-laser and injection. *Gapdh* served as a normalizing control. Data are presented as mean ± SEM, n = 6 for each treatment group. *p < 0.05; **p < 0.005, ***p < 0.0005 isotype vs. DX109. (B) Representative RPE/choroid flat-mounts at day 3 were immunostained with Iba-1, and mean fluorescence intensity (MFI) of Iba-1 calculated from confocal images. Data presented as mean ± SEM, n = 12 lesions for each treatment group; **p < 0.005. (C) Representative RPE/choroid flat-mounts were immunostained with IB4, and mean CNV area calculated from confocal images. Data presented as mean ± SEM, n = 8 lesions for each strain; *p < 0.05.

*In vitro* assessment demonstrates that promoting CD200R signaling with DX109 can directly modulate gene expression of macrophages that are conditioned toward a pro-angiogenic phenotype. We next determined the therapeutic potential of DX109 using the laser-induced CNV model. To this end, DX109 or control isotype mAb were administered to WT mice via intravitreal injection immediately following application of 4 laser lesions to the RPE/choroid. On day 3, the peak time-point for macrophage infiltrate and pro-angiogenic gene expression in this model^18^, RPE/choroid were dissected for analysis of gene expression. In DX109-treated eyes, expression of *Arg-1*, *Ccl2*, *Ccr2* and *Il-1β* in the choroid was significantly reduced compared with choroid from isotype-treated eyes (Figure 5A). The reduction in *Ccr2* expression suggests that DX109 treatment impedes, either directly or indirectly, CCR2^+^ cell recruitment resulting in reduced numbers of pro-angiogenic Arg-1^+^ macrophages to incipient CNV. To determine the degree of macrophage infiltrate, immunohistochemistry with Iba-1 was performed on choroidal flat-mounts derived from DX109- and isotype-treated eyes (Figure 5B). In DX109-treated eyes, there was a significant reduction in levels of Iba-1 expression, reflecting the observed reduction in Ccr2 gene expression. Furthermore, IB4 flat-mount staining revealed that DX109 treatment also resulted in an overall reduction in the total CNV area at this time-point (Figure 5C).

Having demonstrated that promoting CD200R signaling with DX109 treatment leads to reduced proangiogenic gene expression, macrophage infiltration and CNV area, we extended these observations to CD200R-deficient mice. A comparison between WT and *CD200R^−/−^* mice was performed to determine whether the gene signature, in particular the *Arg-1* signal, was also reduced in the choroid of CD200R-deficient animals following treatment with DX109 (Supplementary Figure 5). The results confirm that the reduction in gene expression following treatment is CD200R specific, and that targeting CD200R can suppress the *Arg-1* response associated with the initial stages of laser-induced CNV.

**Figure 6:**
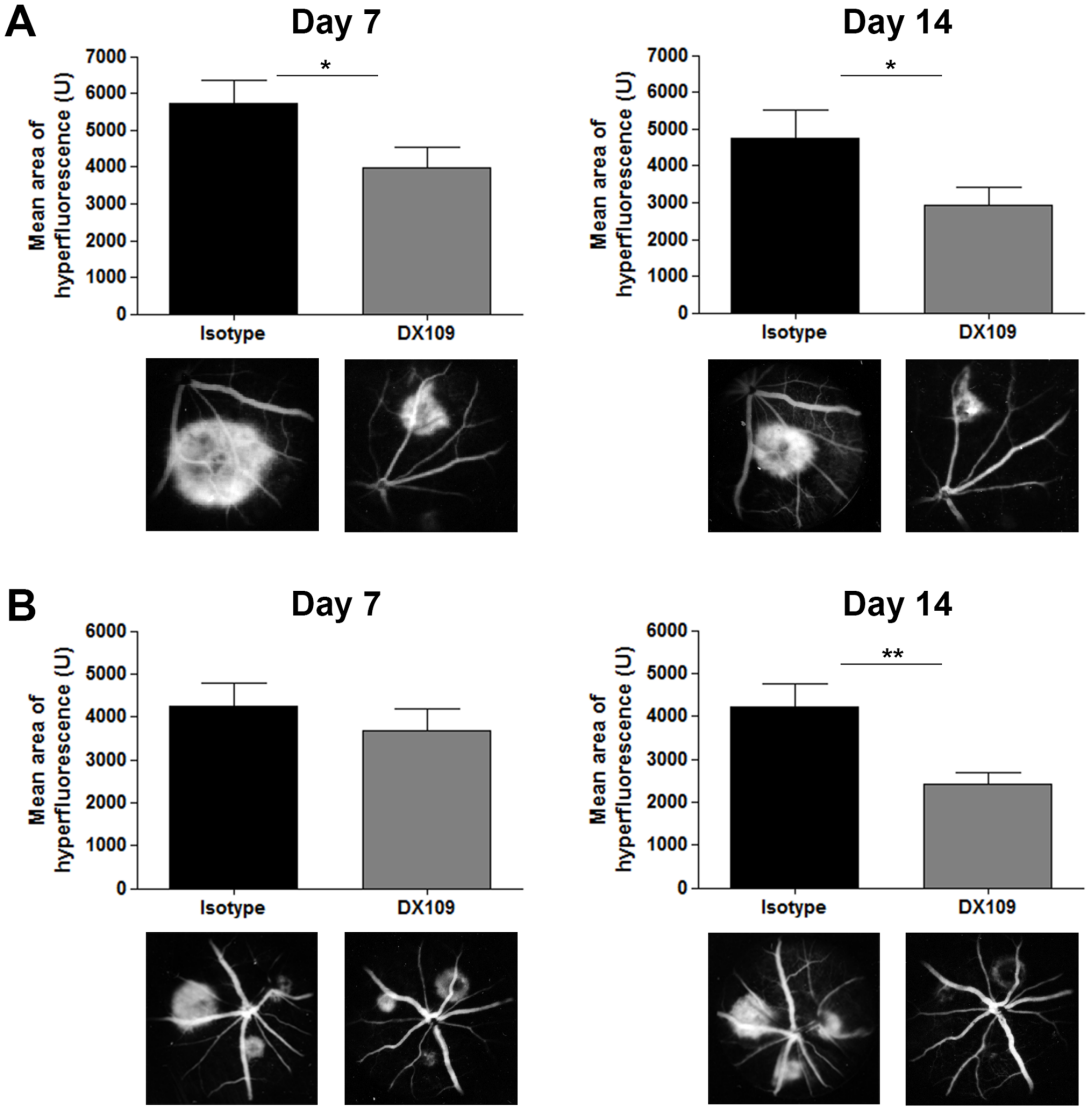
DX109 treatment leads to reduced CNV lesion development. Intravitreal administration of DX109 or isotype control mAb (A) immediately following laser induction of CNV, or (B) at day 3 post-laser in C57BL/6J wild-type mice, and mean lesion size determined by fundus fluorescein angiography performed at 7 and 14 days. Graphs for each time-point show mean lesion sizes following laser and injection of either DX109 or the isotype control mAb. Results are shown from the early phase of fluorescein angiograms (mean area of hyperfluorescence +/− SEM; n = 54 lesions examined), *p < 0.05, **p < 0.005. Representative images from angiograms are shown below each time-point. Mean lesion sizes in mice receiving DX109 compared to isotype were significantly reduced in (A) by 30% and 39% at 7 and 14 days post laser respectively, and (B) by 42% at 14 days.

To establish whether these altered levels of expression and activation of macrophages also impacted the development of CNV, mice were treated immediately following laser injury and lesion size assessed by fundus fluorescein angiography at 7 and 14 days (Figure 6A). The results demonstrate that delivery of the CD200R agonist resulted in a significant reduction in lesion size (39% and 30% at 1 and 2 weeks respectively) compared with isotype-treated eyes. Having demonstrated that manipulating macrophage proangiogenic phenotype early in CNV development attenuates the neovascular response, the question remains as to whether intervention at the time of maximal macrophage infiltration (3 days) post laser could modulate late outcome of CNV. We predicted that intervention at day 3 where macrophage drive of angiogenesis was maximal would not influence the early development of CNV but would suppress in the longer term the extent of CNV. We found that administration of DX109 at day 3 had no apparent impact on CNV at day 7, but resulted in a sustained reduction of 43% in CNV area on fluorescein angiography at day 14 (Figure 6B).

## Discussion

The results of this study demonstrate for the first time that modulation of macrophage phenotype through engagement of CD200R can inhibit proangiogenic function and reduce the extent of pathological angiogenesis in the eye. The laser-induced CNV model used in this study is well established and importantly provides a platform which permits an investigation of the function and behavior of macrophages in the context of an angiogenic drive.

The local administration of a CD200R agonist mAb, known to regulate macrophage function, reveals that targeting early macrophage infiltrate has therapeutic potential to inhibit clinical disease. Indeed our data show that suppression of macrophage activity reduces CNV, but also treating when pro-angiogenic macrophage potential is maximal and VEGF activity is heightened (day 3), results in reduced size of mature lesions. Furthermore, we also report that CD200R deficiency results in augmented macrophage VEGF responses, which generate an increased functional capacity to promote angiogenic HUVEC cell activity, particularly when macrophages are conditioned toward a pro-angiogenic phenotype following stimulation with PGE_2_ or PGE_2_-enriched RPE media *in vitro*. Finally, our data suggest a role for CD200R signaling in maintaining tonic control over VEGF expression, by demonstrating that in absence of CD200R pathological angiogenesis is increased in laser induced CNV. In comparison, *in vitro* assessment demonstrates that promoting CD200R signaling with DX109 directly modulates macrophage gene expression and subverts conditioning with necrotic RPE components toward a pro-angiogenic Arg-1^+^ macrophage phenotype.

Despite an increasing focus on the role of macrophages in retinal and choroidal angiogenesis, the question of how macrophages acquire specific VEGF^+^Arg-1^+^ phenotypes that promote angiogenesis remains unanswered. One notion is that infiltrating myeloid cells are polarized and conditioned by cytokines in the retina and subretinal space adjacent to the RPE. The RPE serves to support the metabolically active photoreceptor layer and maintain ocular homeostasis, not only through visual cycle participation but also by regulating inflammation in the eye^32^,^33^,^34^,^35^. Prostaglandin E2 (PGE_2_) is thought to exert an immunosuppressive effect in the eye and is reported to be secreted in murine primary cultured RPE cells and the human ARPE-19 cell line^24^,^25^. This inflammatory mediator is also recognized to enable alternative activation of macrophages and to promote VEGF expression^12^. In this context, it is conceivable that the RPE is capable of driving a VEGF^+^ macrophage phenotype, and our evidence that both PGE_2_ and PGE_2_-rich RPE cell supernatants induce a VEGF^+^ pro-angiogenic phenotype in macrophages would support this hypothesis^36^,^37^.

The phenotype and functional roles of infiltrating macrophages associated with maintaining vascular integrity and promoting angiogenic responses in the eye are dependent upon how these macrophages are activated^14^. Understanding the complex nature and mechanisms by which multiple drivers, such as senescence^38^, accumulation of lipids or other oxidative stress products^5^,^39^, or damaged components of damaged RPE^18^ influence macrophage function is critical when considering their contribution to ocular neovascularization. Whilst IFN-γ and LPS are factors capable of generating NOS2^+^VEGF^+^M1 macrophages, PGE_2_ remains a potent stimulus for the generation Arg1^+^VEGF^+^ M2 macrophages^12^. Conditioning with other cytokines such as IL-4 or IL-13 still generates Arg-1^+^ M2 macrophages, but these cells express reduced levels of VEGF, and instead secrete large quantities of sFlt-1, an anti-angiogenic factor [Wu W-K, personal communication]. Arg-1 is also an important factor that regulates inflammation, with documented involvement in chronic inflammatory conditions and vascular dysfunction in retinopathy^9^,^40^,^41^. In the context of the current study, the consequence of promoting CD200R signaling is to modulate choroidal gene expression, in particular the Arg-1 signal and subsequent CNV development.

The NLRP3 Inflammasome is implicated as having a central role in the pathogenesis of AMD, specifically components of drusen have been shown to activate NLRP3, causing secretion of IL-1β and IL-18^15^. It has been postulated that NLRP3 and IL-18 may have a protective and anti-angiogenic effect in terms of regulating the development of CNV. Interestingly, whilst the current data demonstrates that CD200R^−/−^ macrophages predominantly elicit an enhanced *IL-1β* response, *IL-18* levels were also elevated (albeit to a far lower level) when compared to wild-type. Whether CD200R signaling also contributes to such protective mechanisms and plays a potential role in the wider context of regulating inflammasome activation will require further investigation. Previous studies using the laser-induced CNV model demonstrate the importance of IL-1β as a key driver for angiogenesis in AMD, and approaches to inhibit its activity using IL-1 receptor antagonists can prevent neovascular development^42^. Thus, the observed reduction in IL-1β levels following DX109 treatment further emphasizes the important potential of this approach in modulating macrophage functional responses and their influence on CNV development.

The ability to target inhibitory receptors has emerged as an effective mechanism to modulate and regulate inappropriate immune activation, characteristic of chronic inflammatory and degenerative diseases^22^,^43^,^44^. CD200 and its myeloid receptor CD200R constitute an inhibitory signaling pathway that provides a homeostatic control mechanism that regulates myeloid cell activation^20^,^45^,^46^. Therapeutic targeting of this pathway has proven successful in numerous inflammatory models^23^,^47^,^48^,^49^,^50^ and CD200R expression, originally described as restricted to cells of myeloid lineage including macrophages, microglia, granulocytes and dendritic cells, is now recognized to include a wide range of immune cell subsets including T cells, B cells and NK cells^19^,^51^,^52^,^53^,^54^. CD200 is expressed abundantly within the normal eye, on retinal vascular endothelium and glial fibrillary acidic protein (GFAP)-negative neurons in retina and optic nerve, and on a subpopulation of CD45-positive perivascular and juxtavascular cells^20^,^21^,^54^,^55^. Moreover, CD200 expression on RPE^56^, and vascular endothelial cells^57^, suggests that its interaction with CD200R-expressing myeloid cells, including resident microglia or infiltrating macrophages may serve to regulate local responses. In our study, we noted that a deficiency of CD200R signaling resulted in augmented VEGF expression and pro-angiogenic functions in macrophages *in vitro* (under PGE_2_ enriched conditions) and more pronounced angiogenesis following laser-induced CNV *in vivo*. Taken together, these data suggest that CD200R signaling regulates myeloid cell phenotypes and consequently ocular neovascularization, specifically via the inhibition of VEGF expression from infiltrating macrophages. Despite *in vivo* targeting of early macrophage infiltrate with DX109 influencing pro-angiogenic gene expression, macrophage recruitment and subsequent CNV development (Figures 5 & 6), we were unable to directly demonstrate *in vitro*, CD200R-mediated suppression of RPE-induced *Vegfa* expression (Supplementary Figure 4). Whether this remains as VEGF independent suppression is still very interesting in itself and may add weight to considering combinatorial approaches in the future for therapy. Therefore manipulation of the CD200-CD200R pathway may prove a beneficial adjunct in the treatment of neovascular AMD.

The monoclonal antibody DX109, originally generated from investigations detailing the cellular distribution of the inhibitory CD200R protein^52^, was subsequently shown to signal as a functional agonist capable of inhibiting myeloid cell responses^19^,^46^. Experimentally, promoting CD200R-mediated signaling with DX109 has been shown to suppress classical macrophage activation and protect against tissue damage during autoimmune responses in the retina^23^. Here, we provide further evidence of the potential clinical benefits of DX109 application, specifically that early targeting of proangiogenic myeloid infiltrate alters angiogenic development and disease progression, whilst delayed administration, even though it does not circumvent the initial drive, the *in vivo* half-life of DX109 (5 days) is still capable of attenuating the extent of CNV in the latter stages of development. Thus, despite the presence of RPE-derived VEGF and other pro-angiogenic factors, triggering the CD200R still has clinical benefit in terms of the maintenance of CNV.

In addition, it should also be considered that the observed suppressive effects of DX109 treatment may also arise from altered responses of other CD200R-positive immune cells. Histological studies demonstrate macrophages, lymphocytes, and mast cells, as well as fibroblasts, are associated with both atrophic lesions and neovascularization of the retina^58^. Mast cells express CD200R, and receptor specific agonists including DX109, can inhibit their inflammatory function, by blocking degranulation and secretion of inflammatory mediators^19^. Furthermore, recent evidence implicates infiltrating γδT cells, that express IL-17 in response to macrophage- and photoreceptor-derived IL-1β and high-mobility group box 1 (HMGB-1), in CNV development^59^. Whilst IL-17 is generally considered a pro-inflammatory cytokine linked to autoimmunity, it can also promote angiogenesis^60^. In this study, both depletion of γδT cells or suppression of IL-1β and HMGB-1 were shown to ameliorate CNV. The inhibitory effect of DX109 in terms of reducing macrophage and choroidal IL-1β gene expression may therefore indirectly impact γδT cell IL-17 production, or alternatively, since γδT cells express CD200R^61^, the agonist may act directly to suppress activation of this inflammatory cytokine network. Thus, in the current study CD200R receptor-mediated signaling may also regulate mast cell and γδT cell responses, and potentially inhibit their contribution to angiogenesis and cell injury in the laser-CNV model.

In summary, CD200R signaling acts to suppress a PGE_2_- or RPE fragment-induced pro-angiogenic macrophage phenotype and the loss of such regulation leads to more severe ocular neovascularization in *CD200R1^−/−^* mice than WT mice. Harnessing this mechanism, by using the CD200R agonist mAb DX109, attenuates the neovascular response in a model of CNV and indicates a potential novel therapeutic approach to the treatment of neovascular age-related macular degeneration.

## Methods

### Mice

C57BL/6J (CD200R^+/+^) mice and CD200R^−/−^ breeding colonies were established in Animal service unit of University of Bristol. All mice were housed under specific pathogen-free conditions with continuously available food and water. The mice were aged between 6 and 8 weeks. Treatment of animals conformed to the Association for Research in Vision and Ophthalmology Statement for the Use of Animals in Ophthalmic and Vision Research. Experiments were carried out in compliance with University of Bristol institutional guidelines and under the authority of a project licence from the UK Home Office.

### Reagents

The agonist rat IgG1-anti-mouse CD200R mAb (DX109) was generated from rats using immunogenic fusion proteins consisting of the extracellular domains of *CD200R* gene fused to the Fc domain of human immunoglobulin and was a gift from Joe Phillips & Jonathon Sedgwick (SP Biopharma, California, USA). The control isotype mAb used was a rat anti-human IL-4. Both mAb preparations used contained < 0.1 ng of endotoxin/mg of protein, as determined by the Limulus Amebocyte Lysate Pyrogen Testing kit, QCL-1000 (Cambrex, East Rutherford, NJ).

Complete medium consisted of Dulbecco's modified Eagle's medium(DMEM) supplemented with 10% heat inactivated fetal calf serum, 100 U/ml penicillin–streptomycin, 2 mmol/l L-glutamine, 1 mmol/l sodium pyruvate and 5 × 10^−5^ mol/l 2-mercaptoethanol (all from PAA, Yeovil, UK).

### CNV Induction and therapeutic intervention

CNV was induced by laser photocoagulation in mice aged 6–8 weeks. In brief, the pupils of animals were dilated using topical 1% tropicamide and mice anesthetized by intraperitoneal (i.p.) injection of 200 μl of Vetelar and Rompun mixed with sterile water in the ratio 0.6:1:84. Four laser spots were delivered to the posterior retina using an OculightSlx Krypton Red Laser system (power 200 mW, duration 75 ms, spot size 75 μm).

Local administration of DX109 or isotype control mAb was performed by intravitreal injection, either immediately following laser or at day 3. In brief, the eye was proptosed and held in position with a pair of forceps, while 10 μg of mAb/4 μl of PBS was injected using a 33-gauge hypodermic needle, (Esslab, Essex, UK). The injection site was treated with chloramphenicol and globe reposited.

### Cell culture and *in vitro* assays

Bone marrow derived macrophages (BMMΦ) were generated using a previously described method^23^. In brief, bone marrow cells were washed in DMEM media and resuspended at 1 × 10^5^ cells/ml in complete medium supplemented with 5% horse serum (PAA, UK) and 50 pg/ml macrophage-colony stimulating factor (M-CSF). Cell suspensions (50 ml) was transferred to Teflon-coated tissue culture bags (supplied by Dr. M. Munder, University of Heidelberg, Germany) and incubated for 8 days at 37°C in 5% CO_2_. Cells were removed from the Teflon bags and plated at 1 × 10^6^ cells/well in 24-well plates (Corning-Costar, Corning, NY), before stimulation with media alone, PGE_2_ (Sigma-Aldrich, Poole, UK) or B6-RPE07 cell conditioned media for 24 hrs.

For the HUVEC-BMMΦ co-culture assay, HUVEC cells cultured in EGM-2 media (Lonza, Switzerland) were washed and then co-cultured with or without BMMΦ established from WT mice or CD200R1^−/−^ mice, stimulated or unstimulated by PGE_2_ (500 ng/ml). Co-culture was conducted on matrigel (Becton Dickinson, UK) in 96-well plate, containing EBM-2 culture media (Lonza, Switzerland). 6000 HUVECs in a well were co-cultured with 5000 BMMΦ for 6 hrs, and cumulative tube length per field was measured by Image Pro Plus software from bright field microscope images. BMMΦ used for co-culture were cultured with PGE_2_ (500 ng/ml) for 24 hrs in advance in the Teflon coated bag and used directly to co-culture.

For the RPE phagocytosis assay, necrotic RPE was generated from a mouse RPE cell line B6-RPE07^26^ by heating a known number of cells for 15 minutes at 95°C, and cell death confirmed by trypan blue staining. BMMΦ cells were plated at a concentration of 1 × 10^6^ cells/well in 24-well plates and incubated for 2 hours to allow cell attachment. Cell culture supernatant was replaced with serum-free DMEM medium containing 1 × 10^6^ necrotic RPE cells, for additional 24 hours incubation. Cells were then washed before subsequent downstream gene analysis.

### RNA extraction and real time RT-qPCR

Total RNA from RPE/choroid tissue or BMMΦ cell cultures was isolated using TRIzol reagent (Life Technologies). One μg of total RNA was treated with RQ1 RNase-free DNase before reverse-transcription using the ImProm-II™ Reverse Transcription System (Promega). cDNA was amplified using the Power SYBR® Green PCR Master Mix Reagent (Applied Biosystems) on a StepOne™ Real-Time PCR System (Applied Biosystems). Primer sequences for mouse *Gapdh*, *18 s rRNA*, *Arg-1*, and *Vegfa* were described previously^12^,^62^. Primers for other mouse or human genes were designed using the PrimerQuest online tool (http://eu.idtdna.com/Scitools/Applications/Primerquest/): mouse *Il1b*: forward 5′-GCCCATCCTCTGTGACTCAT-3′; reverse 5′-AGGCCACAGGTATTTTGTCG-3′; *Ccr2*: forward 5′-AATGAGAAGAAG AGGCACAGGGCT; reverse 5′-ATGGCCTGGTCTAAGTGCTTGTCA-3′; Ccl2: forward 5′- TCACCTGCTGCTACTCATTCACCA-3′; reverse 5′- TACAGCTTCTTTGGGACACCTGCT-3′.

### Cytokine measurements

Cytokine production (VEGF and IL-1β) in culture supernatants from *in vitro* PGE_2_ stimulated BMMΦ, and PGE_2_ concentration of RPE-conditioned media, were assessed by ELISA, following manufacturer's instructions (R&D Systems, UK). Cultured supernatants were immediately frozen after collection and stored at −20°C until use.

### RPE/Choroid whole mount and BMMΦ confocal microscopy

Eyes were enucleated and fixed in 2% (wt/vol) paraformaldehyde (PFA). After dissection of retina and RPE/choroid, tissues were blocked and permeabilized in 5% BSA, 5% goat serum with 0.3% Triton X-100 in PBS for 2 hours, followed with incubation of primary antibodies (biotinylated IB4; Sigma-Aldrich: AF-488 rat anti-mouse CD11b; Becton Dickinson: Rabbit polyclonal anti-Iba1; Wako Chemicals) in 1% BSA with 0.15% Triton X-100 at 4°C. After thorough washing, samples were incubated with secondary antibodies (AF-488 goat anti-rabbit IgG; Life Technologies: Streptavidin Rhodamine RedX, Jackson ImmunoResearch Labs) at RT for 3 hours in the dark. Tissues were washed and flat-mounted in Vectashield antifade medium (Vector Laboratories) and examined using a Leica TCS-SP2-AOBS confocal laser scanning microscope. Isotype antibodies or negative controls with primary antibody omitted did not show significant fluorescence signal.

BMMΦ cells cultured on glass coverslips with RPE-conditioned media for 24 hrs were washed and fixed in 2% PFA for 2 hrs, blocked and permeabilized in 5% BSA, 5% goat serum with 0.3% Triton X-100 in PBS for 30 mins, before overnight incubation with chicken polyclonal anti-VEGF antibody (1:100; Abcam, Cambridge, UK) 1% BSA with 0.15% Triton X-100 at 4°C. After washing, cells were further incubated with a secondary antibody, DyLight 650-goat anti-chicken IgY (1:400; Abcam, Cambridge UK) for 2 hours in the dark, washed and mounted in DAPI containing Vectashield antifade medium and examined using a SP5-AOBS confocal laser scanning microscope.

### Statistical analyses

Data was analyzed with unpaired Student's t-test (GraphPad Prism software, San Diego, CA). Data are generated as mean ± SEM and representative of at least 2 independent experiments. Values were considered statistically significant at *p < 0.05, **p < 0.005, ***p < 0.0005.

## Author Contributions

D.A.C., S.H., S.J.R. and A.D.D. conceived and designed the experiments. D.A.C., S.H., S.J.R., J.L. and W.W. performed the experiments. Data was analyzed by D.A.C., S.H., S.J.R., R.R.A., J.W.B., L.B.N., M.M., A.D.D. and the manuscript written by D.A.C., S.H., S.J.R., A.D.D. All authors reviewed the manuscript.

## Supplementary Material

Supplementary Information Supplementary figures
