# Supplementary material for: CD200R signaling inhibits pro-angiogenic gene expression by macrophages and suppresses choroidal neovascularization
Source: Sci Rep. 2013 Oct 30;3:3072. doi: 10.1038/srep03072 (PMC3812658; doi:10.1038/srep03072)
Supplement: Supplementary Information — Supplementary figures [file srep03072-s1.pdf]

Supplementary Figures

Manuscript:

CD200R signaling inhibits pro-angiogenic gene expression by macrophages and suppresses choroidal neovascularization

*Shintaro Horie[1,2], *Scott J Robbie[3], Jian Liu[1], Wei-Kang Wu[1], , Robin R Ali[3], James W Bainbridge[3], Lindsay B Nicholson[1,4], Manabu Mochizuki[2], **Andrew D Dick[1,4] and *David A Copland[1]

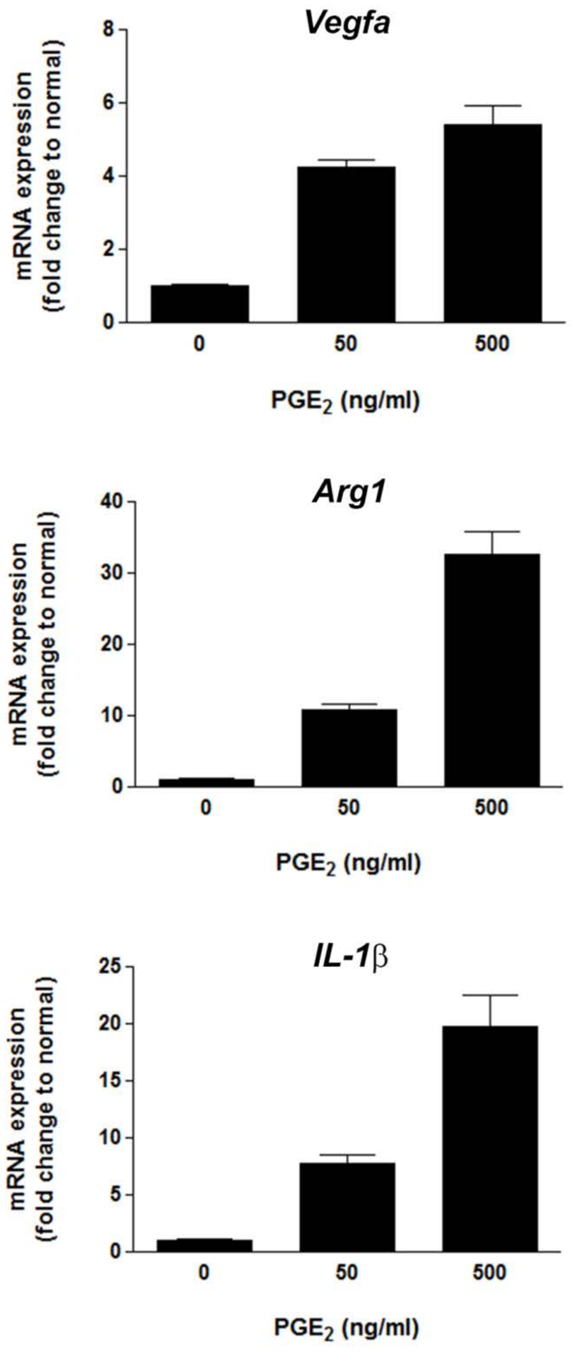

Supplementary Figure 1: PGE_2 induces alternatively activated macrophages

Vegf, Arg1 and Il1b mRNA expression as determined by quantitative RT-qPCR from WT and macrophages stimulated with media alone, 50- or 500- ng/ml PGE_2 for 6 h. Messenger RNA level was normalized to 18srRNA. Data are presented as mean ± SEM, n=3.

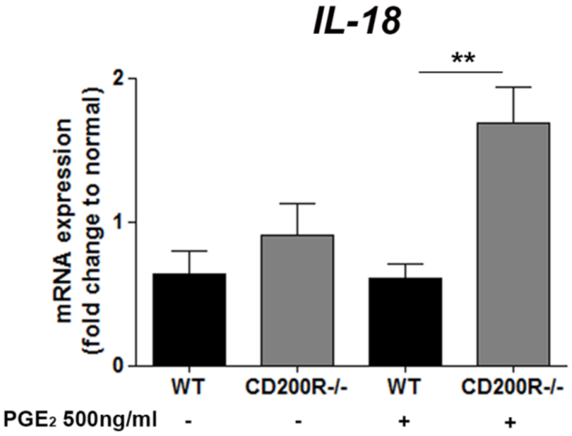

Supplementary Figure 2: CD200R-deficient macrophages demonstrate increased IL-18 expression following PGE_2 stimulation

IL-18 mRNA expression, derived from WT and *CD200R*$^{-/-}$ macrophages stimulated by PGE_2 for 6 h, determined by quantitative RT-PCR. Messenger RNA level was normalized to 18srRNA. Data are presented as mean ± SEM, n=3. **P<0.005 between two groups.

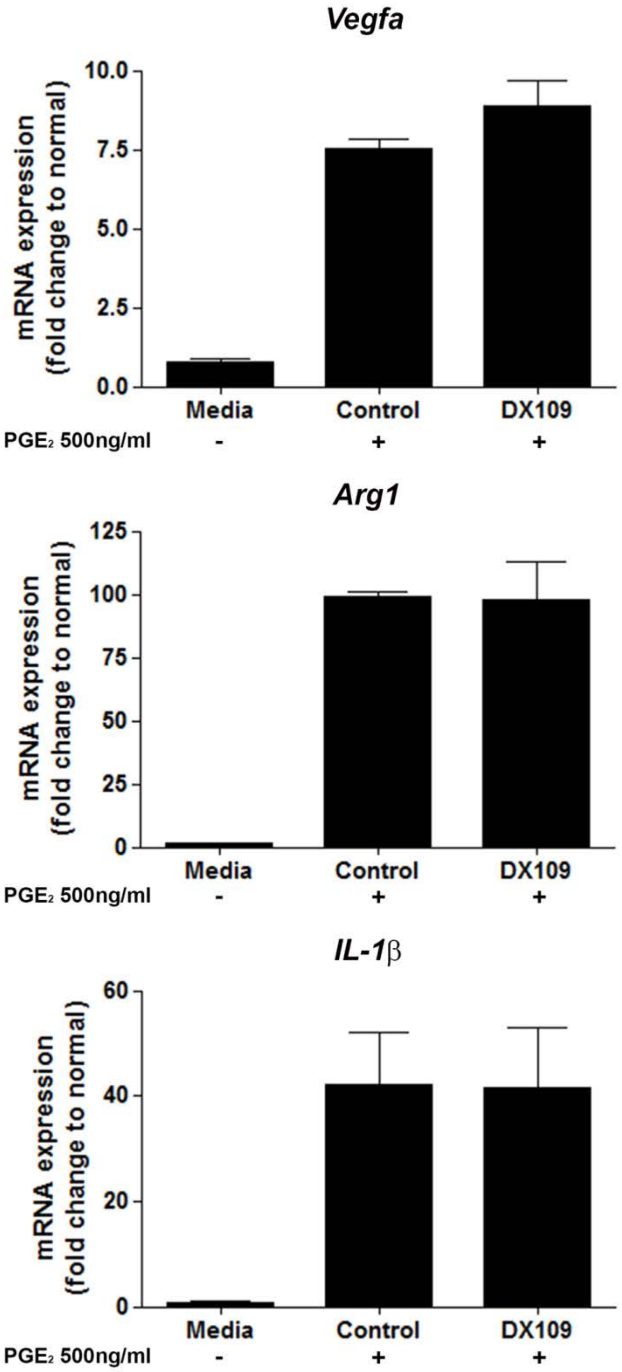

Supplementary Figure 3: Promoting CD200R signaling does not suppresses PGE_2-induced pro-angiogenic gene expression profile of macrophages

BMMΦ were pre-incubated for 2 hours with or without 10 μg/ml DX109 before stimulation with 500 ng/ml PGE2 for 6 hr. RNA was extracted for RT-qPCR analysis to determine *Vegfa*, *Arg-1* and *Il-1b* gene expression. *Gapdh* was used as a normalizing control. Data are presented as mean ± SEM, n=3.

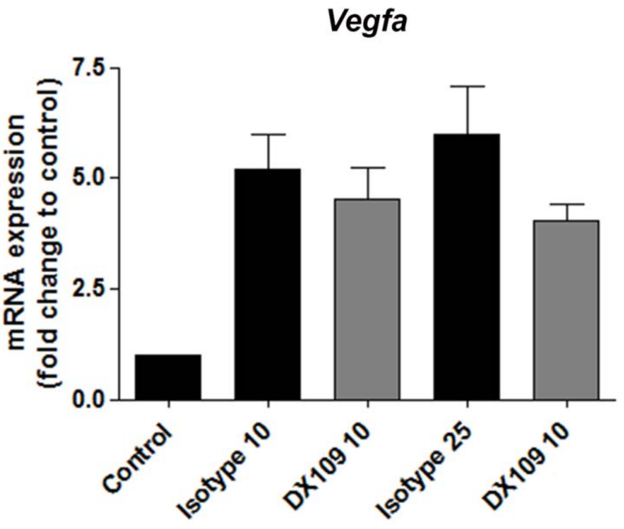

Supplementary Figure 4: Promoting CD200R signaling does not suppress necrotic RPE-induced *vegf* gene expression

Using an adapted *in vitro* phagocytosis assay, necrotic RPE cells prepared from B6-RPE07 cells by heating at 95°C for 15 minutes, were added to BMMΦ that were pre-incubated for 2 hours with 10- or 25 μg/ml DX109 or isotype control mAb. BMMΦ were washed and collected after 24 hr incubation with necrotic RPE, and RNA extracted for RT-qPCR analysis to determine *Vegfa* gene expression. *Gapdh* was used as a normalizing control. Data are presented as mean ± SEM, n=3.

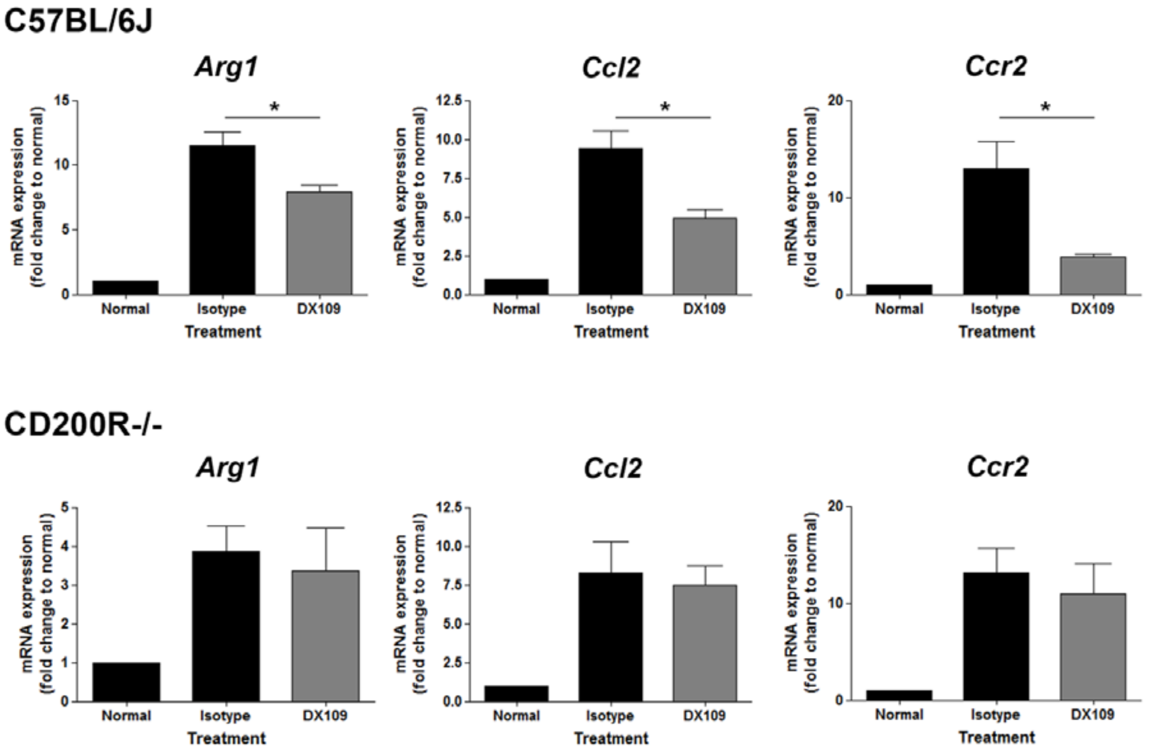

Supplementary Figure 5: Reduction in proangiogenic gene expression is CD200R-specific

Intravitreal administration of DX109 or isotype control mAb was performed immediately following laser induction of CNV in C57BL/6J and *CD200R*$^{-/-}$ mice. RT-qPCR analysis for *Arg-1*, *Ccl2*, *Ccr2* and *Il-1b* gene expression in RPE/choroid tissue collected at day 3 post-laser and injection. *Gapdh* served as a normalizing control. Data are presented as mean ± SEM, n=6 for each treatment group. *$p<0.05$ isotype vs. DX109.
